# Collusion of α-Synuclein and Aβ aggravating co-morbidities in a novel prion-type mouse model

**DOI:** 10.1186/s13024-021-00486-9

**Published:** 2021-09-09

**Authors:** Grace M. Lloyd, Jess-Karan S. Dhillon, Kimberly-Marie M. Gorion, Cara Riffe, Susan E. Fromholt, Yuxing Xia, Benoit I. Giasson, David R. Borchelt

**Affiliations:** 1 Department of Neuroscience, College of Medicine, University of Florida, Gainesville, Florida 32610 USA; 2 Center for Translational Research in Neurodegenerative Disease, College of Medicine, University of Florida, Gainesville, Florida 32610 USA; 3 McKnight Brain Institute, College of Medicine, University of Florida, BMS J499, J483/CTRND, 1275 Center Drive, Gainesville, FL 32610 USA

**Keywords:** Alzheimer’s disease, Aβ, Lewy body dementia, α-Synuclein, Prion-like propagation

## Abstract

**Background:**

The misfolding of host-encoded proteins into pathological prion conformations is a defining characteristic of many neurodegenerative disorders, including Alzheimer’s disease, Parkinson’s disease, and Lewy body dementia. A current area of intense study is the way in which the pathological deposition of these proteins might influence each other, as various combinations of co-pathology between prion-capable proteins are associated with exacerbation of disease. A spectrum of pathological, genetic and biochemical evidence provides credence to the notion that amyloid β (Aβ) accumulation can induce and promote α-synuclein pathology, driving neurodegeneration.

**Methods:**

To assess the interplay between α-synuclein and Aβ on protein aggregation kinetics, we crossed mice expressing human α-synuclein (M20) with APPswe/PS1dE9 transgenic mice (L85) to generate M20/L85 mice. We then injected α-synuclein preformed fibrils (PFFs) unilaterally into the hippocampus of 6-month-old mice, harvesting 2 or 4 months later.

**Results:**

Immunohistochemical analysis of M20/L85 mice revealed that pre-existing Aβ plaques exacerbate the spread and deposition of induced α-synuclein pathology. This process was associated with increased neuroinflammation. Unexpectedly, the injection of α-synuclein PFFs in L85 mice enhanced the deposition of Aβ; whereas the level of Aβ deposition in M20/L85 bigenic mice, injected with α-synuclein PFFs, did not differ from that of mice injected with PBS.

**Conclusions:**

These studies reveal novel and unexpected interplays between α-synuclein pathology, Aβ and neuroinflammation in mice that recapitulate the pathology of Alzheimer’s disease and Lewy body dementia.

**Supplementary Information:**

The online version contains supplementary material available at 10.1186/s13024-021-00486-9.

## Background

Many proteins can enter an amyloidogenic state, wherein a protein, prompted by the surrounding milieu, cellular signaling, or even another protein, adopts a β-sheet structure. These β-sheets can stack upon one another into fibrils stabilized by hydrogen bonding [1] and with time, may accumulate progressively into larger aggregates. Proteins that are able to pass this alternative structure onto naïve copies of themselves through conformational templating are termed prions and are considered to be transmissible agents [2]. Prions, and prion-like proteins, are areas of intense research as their mechanisms of misfolding and propagation, resulting in progressive amyloid accumulation, can lead to neurodegeneration. Neurodegenerative diseases are histologically hallmarked by the fibrous lesions formed from aggregated amyloids, and often exhibit co-pathology of multiple priogenic proteins [3] resulting in a spectrum of proteinopathies categorized by their clinical and histopathological features, rather than just the type of aggregated protein.

Two of the most common forms of age-related neurodegenerative disorders, Alzheimer’s disease (AD) and Parkinson’s disease (PD), which canonically exhibit accumulations of amyloid β (Aβ) and α-synuclein (αSyn) respectively, often exhibit co-pathology of these proteins and represent keystone components on the spectrum of neurodegenerative disorders [4–18]. Lewy body dementia (LBD) oscillates between these disorders, exhibiting both Aβ and αSyn pathology [11, 15, 19–23]. A significant percentage (20–40%) of patients with PD or LBD present with both abundant αSyn inclusions and Aβ deposits [4–11]. Moreover, αSyn inclusions are frequently observed in brains from patients with sporadic and familial AD, where genetic defects in the *APP*, *PSEN1* and *PSEN2* genes directly affect biological pathways that promote Aβ deposition [12, 24–27].

These pathological findings, and the overlap of clinical symptoms between AD and PD patients [13–16, 18, 28–31], suggest that Aβ and αSyn can collude to induce and enhance pathogenesis, possibly due to their infectious nature as priogenic proteins. In an effort to illuminate the interactions between the pathogenic forms of these proteins, we developed a mouse model in which the deposition of Aβ was intrinsically driven by transgenesis, and used intracerebral prion-like seeding to induce αSyn inclusion pathology, as has been previously shown effective in other models of neurodegeneration [32–37]. We used APPswe/PS1dE9 (L85) as our Aβ mouse model; this model develops Aβ deposition by 4–6 months of age [38, 39]. To model interactions between human Aβ and human αSyn, we crossed the L85 mice to the M20 model, which expresses WT human αSyn [40, 41]. M20 mice do not present with an aberrant phenotype or display any αSyn pathology during their normal lifespan [40, 41], but develop extensive αSyn pathology following intracerebral injection of αSyn pre-formed fibrils (PFFs) [36, 37]. Using this model, we investigated the impact that pre-existing Aβ pathology has on the induction of αSyn inclusion pathology by injection of PFF, how αSyn pathology in turn alters Aβ plaque formation, and the interplay of neuroinflammation induced by these pathologies.

## Methods

### Mouse lines

All procedures were performed according to the National Institute of Health Guide for the Care and Use of Experimental Animals and were approved by the University of Florida Institutional Animal Care and Use Committee. Mice were housed in a stable environment with a 12-h light/dark cycle and access to food and water ad libitum. Transgenic mice expressing WT human αSyn (line M20), were generated using the mouse PrP vector (MoPrP.Xho) to drive expression [40, 41], and were maintained on a C57BL/C3H background as hemizygous mice (M20^+/−^) by mating with non-transgenic (nTg) C3H/BL6 (Charles River) mice. APPswePS1dE9 double-transgenic mice (L85) express a chimeric mouse/human amyloid precursor protein (APP), containing known familial mutations in APP (KM670/671NL) and a human presenilin-1 variant carrying the exon 9 deletion, [38, 39]. Both transgenes were expressed together under control of the mouse prion protein promoter (MoPrP.Xho), directing expression predominantly in neurons but also in astrocytes of the CNS [42]. To generate all of the mice used in these studies, M20^+/−^ and L85^+/−^ mice were mated to produce non-transgenic (nTg), M20^+/−^ (M20), L85^+/−^ (L85) and M20^+/−^/L85^+/−^ (dTg) litter mates.

#### Recombinant human αSyn expression, purification and fibril formation

The pRK172 bacterial expression vector containing the cDNA encoding WT human αSyn was transformed into BL21 (DE3)/RIL *E. coli* (*E. coli*; Agilent Technologies) and recombinant αSyn was purified from *E. coli* using size exclusion chromatography followed by anion exchange as previously described [43, 44]. Protein concentrations were determined by bicinchoninic acid assay using bovine serum albumin as the protein standard.

To generate PFFs for injection, recombinant human αSyn protein [5 mg/ml in sterile phosphate buffered saline (PBS)] was incubated at 37 °C with constant shaking at 1050 RPM (Thermomixer R, Eppendorf) for > 48 h. Fibril formation was monitored by K114 [(trans, trans)-1-bromo-2,5-bis-(4-hydroxy) styrylbenzene] fluorometry as previously described [45]. Fibrils were diluted to 2 mg/ml in sterile PBS and sonicated in a water bath for 2 h. Sonicated fibrils were then aliquoted, stored at − 80 °C and thawed when required. Each experiment in this study was performed using PFFs from the same preparation, in order to limit batch to batch variation.

#### Stereotaxic brain injections of αSyn PFFs

nTg, M20^+/−^, L85^+/−^ and dTg mice were injected unilaterally into the hippocampus (coordinates from Bregma: anterior/posterior − 2.2 mm, lateral − 1.6 mm, dorsal/ventral − 1.2 mm) at 6 months of age as previously described [34]. For injection, 2 μL of solution (sterile PBS), containing 4 μg of PFFs was utilized. An additional set of mice in each cohort were injected in the same location with 2 μL of vehicle (sterile PBS) as negative controls. Mice were aged until 8 or 10 months, then were sacrificed for histologic analysis.

#### Tissue processing

At designated time points, mice were euthanized with CO_2_ and perfused with a heparin/PBS solution. For histopathology, brains and spinal cords were harvested and fixed in 70% EtOH/150 mM NaCl, paraffin embedded, and sectioned as previously described [46]. For biochemical analysis, some brains were snap frozen on dry ice and stored at − 80 °C for tissue analysis. The number of animals analyzed and their genotypes, are summarized in Table 1.

**Table 1:** Summary of mice used for studies. Organized by genotype, sex and injection cohort used in the studies

Injection Type/Age	nTg		M20		L85		dTg	
**PBS/10 months (4 m.p.i.)**	4 M	4F	4 M	2F	4 M	4F	2 M	4F
**PFF/8 months (2 m.p.i.)**	4 M	4F	4 M	4 M	4 M	4F	4 M	4F
**PFF/10 months (4 m.p.i.)**	5 M	8F	4 M	8F	4 M	6F	5 M	6F

#### Immunohistochemistry and immunofluorescence

Tissue sections were rehydrated with xylenes and graded, 100–70% ethanol steps [47], followed with only heat-induced epitope retrieval (HIER) in a steam bath for 60 min in water with 0.05% Tween-20, unless otherwise indicated. After antigen retrieval, sections were washed in running deionized H_2_O for 15 min. Endogenous peroxidase was quenched by incubating sections in 1.5% hydrogen peroxide/0.005% Triton-X-100 diluted in PBS, pH 7.4 (Invitrogen) for 10 min. Sections were then rinsed in running deionized H_2_O for 15 min, washed three times for 5 min in 0.1 M Tris, pH 7.6, and then blocked in 2% fetal bovine serum (FBS)/0.1 M Tris, pH 7.6 solution for 5 min. Slides were incubated with primary antibodies diluted in blocking solution and stored overnight in 4 °C. Primary antibodies and dilution factors are listed in Tables 2 and 3.

**Table 2:** List of antibodies used with dilutions and conditions

**Immunocytochemistry /Immunofluorescence**				
**1°Antibody (1°Ab)**	**Dilution**	**Specificity**	**Host**	**Antigen Retrieval**
**81A**	1:1000	αSyn at pSer129	Mouse	Water boil w/ 0.05% Tween
**2H6**	1:5000	αSyn (2–21)	Mouse	Water boil w/ 0.05% Tween
**EP1536Y**	1:1000	αSyn at pSer129	Rabbit	Water boil w/ 0.05% Tween
**5G4**	1:1000	oligomeric, aggregated αSyn (44–57)	Mouse	Water boil w/ Citrate/70% Formic acid
**AB5**	1:1000	Aβ	Mouse	70% Formic acid/Water boil w/ Citrate
**12F4**	1:500	Aβ_1–42_	Mouse	70% Formic acid/Water boil w/ Citrate
**33.1.1**	1:500	Aβ_1–16_	Mouse	70% Formic acid/Water boil w/ Citrate
**13.1.1**	1:800	Aβ_1–40_	Mouse	70% Formic acid/Water boil w/ Citrate
**GFAP (from Dako)**	1:2000	Astrocytes, glial cells	Rabbit	Water boil w/ 0.05% Tween
**Iba1**	1:1000	Macrophages, microglia	Rabbit	Water boil w/ Citrate/70% Formic acid
**p62**	1:2000	Sequestresome1	Rabbit	Water boil w/ 0.05% Tween
**NFL**	1:500	NFL	Rabbit	Water boil w/ Citrate
**Western blotting**				
**1°Antibody (1°Ab)**	**Dilution**	**Epitope**		**Host**
**94-3A10**	1:1000	human and mouse αSyn (130–140)		Mouse
**6E10**	1:1000	Aβ		Mouse
**15-4A5**	1:1000	human αSyn (120–125)		Mouse
**C4**	1:1000	actin		Mouse

**Table 3:** Key Resources

**Key Resources Table**		
**Antibodies**	**Source**	**Identifier**
pSer129 αSyn	B. Giasson University of Florida College of Medicine; Florida; USA	81A [48]
2–21 mouse and human αSyn	B. Giasson University of Florida College of Medicine; Florida; USA	2H6 [49]
130–140 mouse and human αSyn	B. Giasson University of Florida College of Medicine; Florida; USA	94-3A10 [49]
120–125 human αSyn	B. Giasson University of Florida College of Medicine; Florida; USA	15-4A5 [49, 50]
pSer129 αSyn	Abcam	EP1536Y [51]
1–16 Αβ	T. Golde University of Florida College of Medicine; Florida; USA	Ab5 [52]
4–10 Αβ; APP	Biolegend	6E10; Cat # 803003
x-42 Αβ specific	EMD Millipore Corporation, Temecula, CA	12F4; Lot: 3270770
x-40 Α specific	T. Golde University of Florida College of Medicine; Florida; USA	13.1.1 [52]
1–16 Αβ	T. Golde University of Florida College of Medicine; Florida; USA	33.1.1 [52]
GFAP	Dako, Carpentaria, CA	GFAP Dako Cat # Z0334
p62/sequestresome1	ProteinTech, Rosemont, IL	
NFL	Cell Signaling	Cat# C28E10
5G4	Fisher Scientific	Cat # MABN389MI [53]
Actin	Fisher Scientific	Clone C4; Cat# MAB1501MI
**Experimental Models: Organisms/Strains**		
**Cohort Name**	**Source**	**N**
Line 85	D. Borchelt University of Florida College of Medicine; Florida; USA	M:12; F:14
M20	B. Giasson University of Florida College of Medicine; Florida; USA	M:12; F:14
dTg	D. Borchelt University of Florida College of Medicine; Florida; USA	M:11; F:14
C3H/BL6 (nTg)	Charles River	M:13; F:16
**Chemicals, peptides, and Recombinant Proteins**		
Human α-synuclein PFFs	This manuscript	N/A
**Software and Algorithms**		
Prism 7	GraphPad	

After overnight incubation, primary antibody was removed from slides with a quick rinse, then incubated with agitation for 5 min in 0.1 M Tris, pH 7.6, three times. Tissue sections were incubated for 1 h with either goat anti-rabbit or anti-mouse biotinylated IgG (Vector Laboratories; Burlingame, CA) in 0.1 M Tris, pH 7.6/2% FBS at room temperature. Secondary antibody was rinsed three times with 0.1 M Tris, pH 7.6 for 5 min. Sections were then incubated with an avidin-biotin complex (ABC) solution (Vectastain ABC Elite kit; Vector Laboratories, Burlingame, CA) for 1 h at room temperature, then rinsed again, three times, with 0.1 M Tris, pH 7.6, for 5 min. Sections were developed using chromogen 3,3′-diaminobenzidine (DAB kit; KPL, Gaithersburg, MD) and counterstained using hematoxylin (Sigma Aldrich, St. Louis, MO). For Aβ immunohistochemistry (IHC), optimized epitope unmasking and antigen retrieval was performed using methods previously described [54]. Succinctly, sections were treated with 70% formic acid for 10 min at room temperature, treated in a steam bath in 0.05% Tween-20 and modified citrate buffer (Target Retrieval Solution Citrate pH 6; Agilent, Santa Clara, CA) for 30 min, and cooled to room temperature for 30 min. Rinsing, blocking and primary dilution steps remain congruent with standard methods. For secondary antibody application, ImmPRESS polymer secondary antibody (Vector Laboratories; Burlingame, CA) was applied to sections for 90 min at room temperature; DAB solution was warmed to 37 °C prior to application. For ionized calcium binding adaptor molecule 1(Iba1) IHC, sections were incubated in formalin for 48 h after rehydration. Sections were then rinsed in water for 10 min. HIER was performed for 30 min using modified citrate buffer (Target Retrieval Solution Citrate pH 6; Agilent, Santa Clara, CA), then treated with 70% formic acid for 10 min. Rinsing, blocking and primary dilution steps remain the same as indicated above. For secondary antibody application, ImmPRESS polymer secondary antibody (Vector Laboratories; Burlingame, CA) was diluted in a 1:10 ratio with the standard secondary antibody solution described above. The remainder of this protocol is same as described above.

Double labeling with rabbit anti-neurofilament light chain (NFL) (C28E10; Cell Signaling Technology) followed the rehydration steps described above, with HIER in a steam bath for 60 min using modified citrate buffer. Rinsing, blocking and primary dilution steps remain the same as indicated above. For secondary antibody application, the previously described DAB reaction was first completed. Tissue was then rinsed and ImmPRESS anti-rabbit conjugated to alkaline phosphatase (Vector Laboratories) was applied for 90 min. After washes, sections were incubated in 0.1 M Tris, pH 8.45 for 30 min, and labeling was visualized with Vector Red substrate (Vector Laboratories). Tissue sections were then counterstained with hematoxylin, dehydrated and mounted as described above.

For immunofluorescence analysis (IFA), antigen retrieval was performed using standard methods with the following modifications: quenching endogenous peroxidase was not performed, primary antibody was diluted in 5% skim milk/TBS. Following incubation overnight with primary antibodies, and previously described rinsing method, tissue sections were incubated with Alexa Fluor 488 or 594-conjugated secondary antibodies (Invitrogen, USA) for 2 h at room temperature, then washed in 0.1 M Tris, pH 7.6 for 20 min. To reduce background lipofuscin autofluorescence, sections were incubated in a 0.3% Sudan Black/70% ETOH solution for 10 min at room temperature, then rinsed in deionized H_2_O for 5 min. In order to stain the nuclei, slides were incubated for 5 min in 4,6 diamidino-2-phenylindole (DAPI) stain (1 μg/ml) diluted in PBS. Slides were then washed in deionized H_2_O for 5 min and cover-slipped using Fluoromount-G (Southern Biotech).

#### Semi-quantification and digital analysis of pathology

All IHC sections were digitally scanned using an Aperio ScanScope CS instrument (40× magnification; Aperio Technologies Inc., Vista, CA, USA), and images of representative areas of pathology were captured using the ImageScope software (40× magnification, Aperio Technologies Inc.). Tissue sections stained with the following antibodies: 81A, p62 and AB5, were semi-quantified via manual counting of positively stained inclusions/plaques by two blinded observers at 20x objective. For quantification of gliosis, GFAP and Iba1 stained sections were analyzed using Aperio ImageScope. Regions of interest (ROIs) were selected in the retrosplenial cortex, CA1 of the hippocampus and amygdala/entorhinal cortex and quantified separately. A modified version of ImageScope’s Positive Pixel Count algorithm v9 was used to measure the intensity of individual stains within the selected ROI, classifying them as either ‘Weak’, ‘Medium’, or ‘Strong’. In order to maximize pathology detection and minimize background, statistical analysis was completed using only values classified as ‘Strong’ positivity. For quantification of αSyn pathology, 81A and 5G4 stained sections were analyzed using a modified version of ImageScope’s Color Deconvolution algorithm v9, tailored to each staining, and slides were scored based on the quantified optical density (OD) analysis of the immunoreactive area (IRA) for the DAB color channel. For analysis and input into heatmaps, scores were normalized by setting the highest score from all cohorts as the maximum value. IFA sections were visualized using an Olympus BX51 microscope mounted with a DP71 Olympus digital camera to capture images at 20x/40x magnification. Representative images were adjusted for white/black values; brightness/contrast corrections were applied identically on captured images within each figure using Adobe Photoshop CS3 (Adobe Systems, San Jose, CA, USA). All raw files and algorithms are available upon request.

#### Western blot analysis

Whole mouse brains were quickly frozen on dry ice and stored at − 80 °C before extraction. Tissue from eight mice were thawed, individually sonicated in 4% SDS/50 mM Tris, pH 7.6, and heated for 10 min at 90 °C. Protein concentrations for all fractions were determined using the BCA assay (Pierce, Waltham, MA, USA), using bovine serum albumin as a standard. Samples were normalized for total protein content and SDS-containing sample buffer was added to samples, which were then further heated for 10 min at 90 °C. Protein samples were separated on SDS-polyacrylamide gels (8% or 15%) and transferred electrophoretically onto 0.22 μm nitrocellulose membranes (Bio-Rad, Hercules, CA). Membranes were blocked in 5% non-fat milk in Tris-buffered saline, pH 7.6 (TBS) for 1 h at room temperature, then incubated in primary antibodies (detailed in Table 2) diluted in 5% non-fat milk/TBS block solution overnight in 4 °C. After incubation, membranes were rinsed with agitation in TBS for 5 min, repeated eight times. Membranes were then incubated with goat anti-mouse secondary antibody conjugated to horseradish peroxidase (Jackson Immuno Research Labs, Westgrove, PA), diluted 1:1000 in 5% non-fat milk/TBS for 2 h at room temperature. Protein band signal was detected with Western Lightning-Plus ECL reagents (PerkinElmer, Waltham, MA) and chemiluminescence imaging (PXi, Syngene, Frederick, MD).

#### Statistical analysis

The number of samples or animals (n) analyzed for each experiment, the statistical analysis performed and the *p*-values for all results are reported in the Table 1, Figures, and/or Figure Legends. Data was tested for normality using D’Agostino-Pearson test. A Two-Way ANOVA was used to compare quantified IHC results of PBS and αSyn PFF-injected animals between each cohort; Holm-Sidak test was used to correct for multiple comparisons and each *P* value was multiplicity adjusted. Family-wise significance was set at 0.05. Statistical analysis was performed using Prism software (GraphPad Software, San Diego, CA, USA). Data are presented as mean +/− SEM, and level of significance was set at *p* < 0.05.

## Results

### Antecedent Aβ pathology leads to exacerbation of induced αSyn inclusion formation

**Fig. 1:**
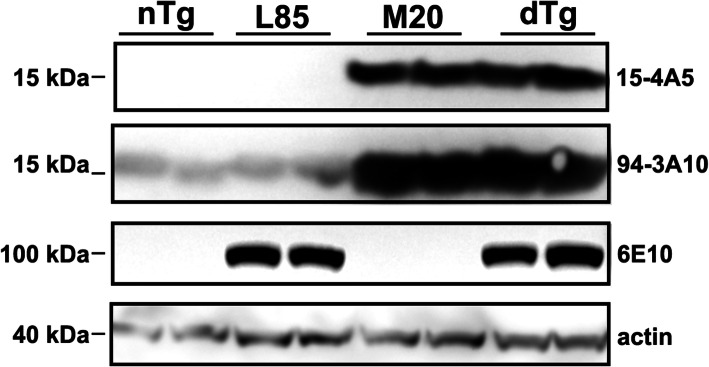
Immunoblots Showing Relative Levels of αSyn and APP. Western blots were conducted on whole brain lysates from 10-month-old, non-injected nTg, L85, M20 and dTg mice. Membranes were probed using antibodies specific for human αSyn (15-4A5), human and mouse αSyn (94-3A10), human APP (6E10) or actin, as indicated

To investigate the interplay between the formation of αSyn inclusion pathology and Aβ deposition, we crossed M20 transgenic mice [40, 41] with L85 mice [38, 39]. Mice harboring the M20 αSyn transgenes and the L85 APPswe/PS1dE9 transgene complexes are hereinafter referred to as ‘dTg’. Overexpression of human αSyn in M20 and dTg mice was confirmed by western blot analysis on whole brain lysate using antibodies specific for human αSyn (15-4A5) and total human and mouse αSyn (94-3A10), respectively (Fig. 1). Similarly, overexpression of APP in L85 and dTg mice was established by western blotting with 6E10 antibody (Fig. 1). Importantly, the levels of overexpression of human αSyn and APP in the original respective mouse line compared to nTg mice was similar (Fig. 1).

**Fig. 2:**
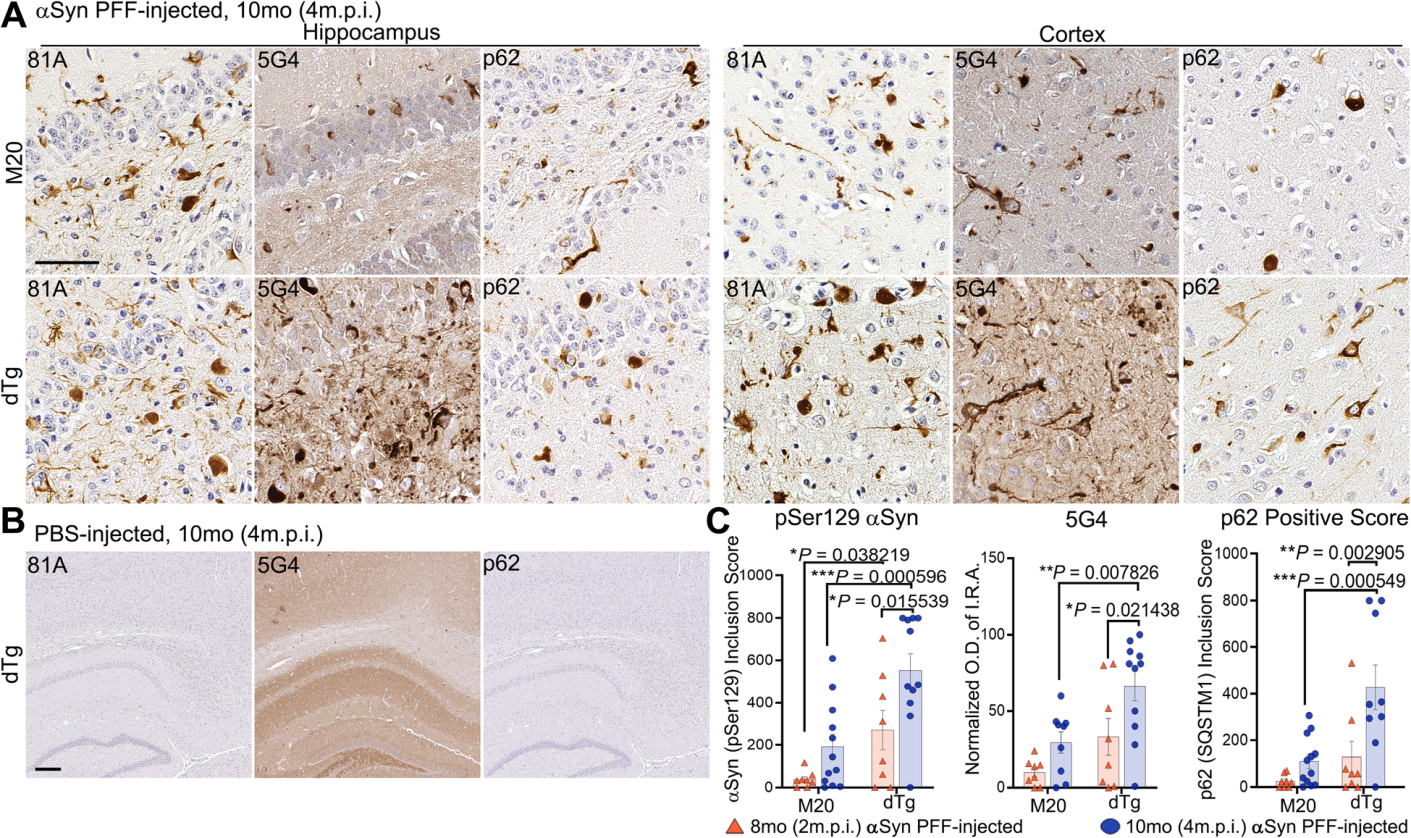
αSyn PFF-Injected dTg Mice Exhibit Exacerbated αSyn Pathology Compared to αSyn PFF-Injected M20 Mice. **A** Representative IHC images of hippocampus and cortex from M20 and dTg mice (10-month-old; 4 m.p.i.) that were injected with αSyn PFFs, and stained with antibodies specific for αSyn phosphorylated on Ser129 (81A), aggregated αSyn (5G4), and p62/sequestrasome-1. **B** Representative images demonstrating the lack of pathological αSyn inclusions in PBS-injected dTg mice (10-month-old; 4 m.p.i.). **C** Semi-quantitative analysis comparing 81A and p62-positive inclusions, or normalized quantitative analysis of OD of 5G4 IRA, between M20 and dTg mice injected with αSyn PFFs. Two-Way ANOVA followed by Holm-Sidak’s multiple comparisons test (*n* = 8, 12; 8,11). Data are presented as mean +/− SEM. Scale bars: (A) 50 μm; (B) 200 μm

Human αSyn PFFs and PBS (control) were stereotactically injected in the hippocampus of 6-month-old mice which were then aged to 8 or 10 months. At 6 months of age, L85 mice have substantial Aβ deposition that would be expected to continue to worsen with age [55, 56]. The number of mice in each cohort are detailed in Tables 1 and 3 and are indicated in each figure legend. nTg and L85 mouse cohorts, which only express endogenous mouse αSyn, did not present with any αSyn inclusion pathology even after the intracerebellar injection of 4 μg of human αSyn PFFs (Supplementary Figure 1). However, both dTg and M20 mice injected with the same PFF preparations exhibited widespread αSyn pathology throughout hippocampal and cortical regions (Fig. 2A). Consistent with previous findings [37], PBS-injection did not elicit αSyn pathology in any cohort, including dTg (Fig. 2B). Analysis with pSer129 antibody 81A, and aggregate specific αSyn antibody, 5G4, revealed that dTg mice present with more abundant αSyn inclusion pathology than M20 mice (Fig. 2A). This finding was also confirmed with an antibody to p62/sequestasome-1, a marker of protein aggregation [57–59]. Semi-quantification of αSyn inclusion pathology stained with pSer129 antibody 81A or p62/sequestrasome-1 in αSyn PFF-injected 8-month or 10-month mice further revealed significantly more pathology in dTg mice compared to M20 mice (Fig. 2C). Quantification IRA OD for 5G4 staining revealed a significant increase in aggregated αSyn in dTg mice when compared to age-matched M20 mice, as well as an increase within the dTg cohort with age (Fig. 2C).

**Fig. 3:**
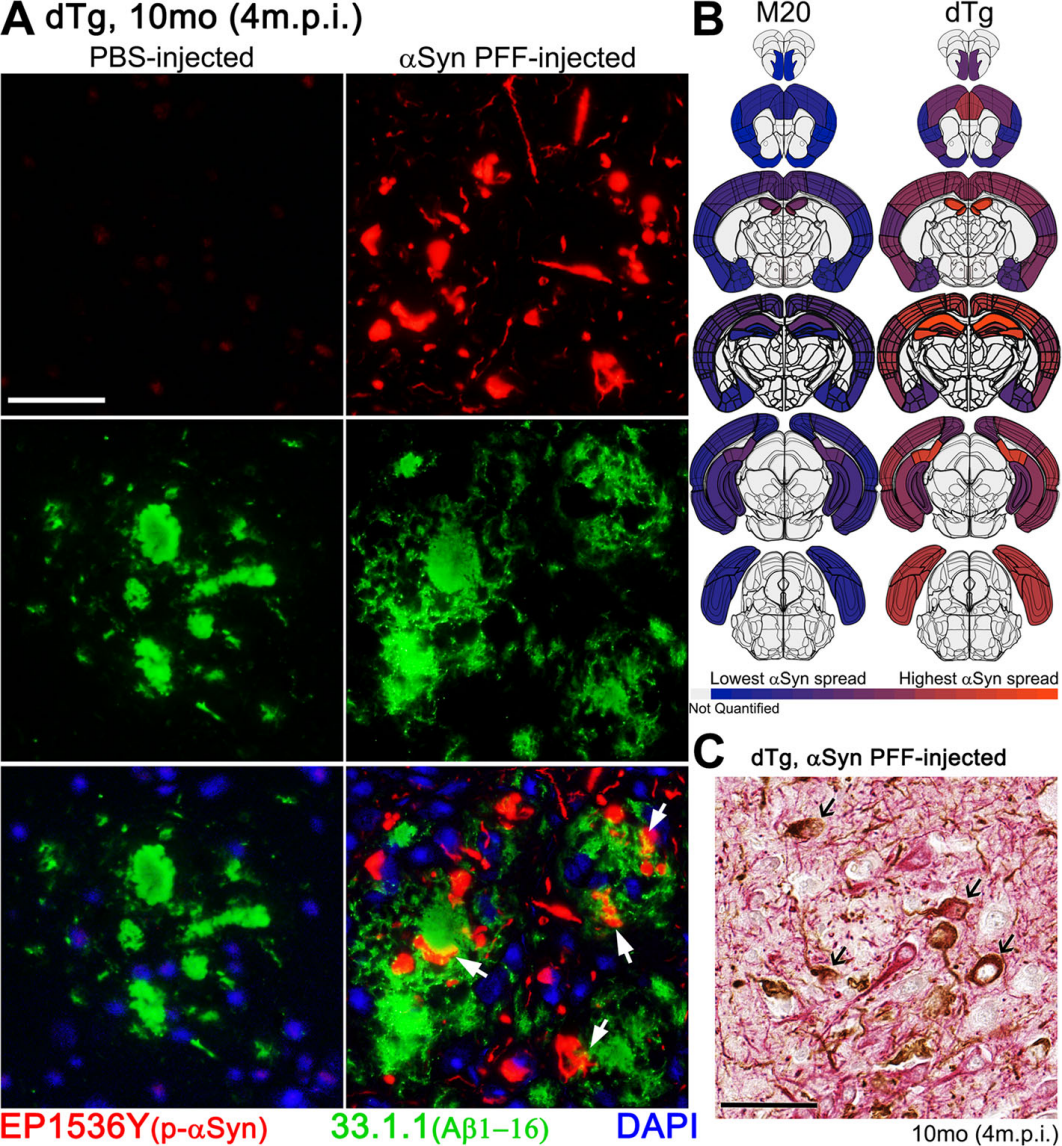
Phosphorylated αSyn-Positive Inclusions Contiguous to Cored Aβ Plaques. **A** IFA double labeling of phosphorylated αSyn-positive inclusions (EP1536Y; red) and Aβ (1–16) plaques (33.1.1; green) in the cortex, comparing age-matched dTg mice (10-month-old; 4 m.p.i.) injected with PBS or αSyn-PFFs. White arrows depict αSyn inclusions in close proximity to Aβ plaques. **B** Regional comparison of phosphorylated αSyn-positive pathology between age-matched, αSyn PFF-injected M20 and dTg cohorts. The level of αSyn pathology is illustrated by the color change from blue (minimum of total counted 81A positive inclusions) to orange (maximum of total counted 81A positive inclusions). Light gray indicates regions were not quantified during this study. **C** Double staining of αSyn PFF-injected 10-month-old (4 m.p.i.) dTg mice with anti-NFL (red) and anti-αSyn 81A (brown) antibodies in the cortex. Arrows indicate neuronal cell bodies labelled for NFL and positive for αSyn inclusions. Scale bars: 50 μm

Induced αSyn inclusion pathology was often, but not exclusively, present in close proximity to Aβ plaques in profiles resembling swollen neurites (arrows in Fig. 3A). The overall distribution of αSyn pathology was modified in the presence of Aβ, with a dramatic increase in pathology both anterior to and posterior of the injection site (Fig. 3B). These findings indicate that the hippocampal injection of αSyn PFFs in dTg mice produces a more rapidly spreading αSyn pathology than what occurs in mice expressing only human WT αSyn.

In both M20 and the dTg mice, a significant portion of αSyn pathology was localized to the neuropil where it is difficult to discern cellular origin. To further characterize pathology in the cortex, we co-stained sections from the dTg mice with antibodies to the neurofilament light chain (NFL) subunit (a neuronal marker) and αSyn (Fig. 3C). The majority of the induced αSyn pathology in cell bodies was present in neurons and the preponderance of αSyn neurites were also labeled for NFL (Fig. 3C).

### Induction of pathological αSyn inclusions affects Aβ deposition

**Fig. 4:**
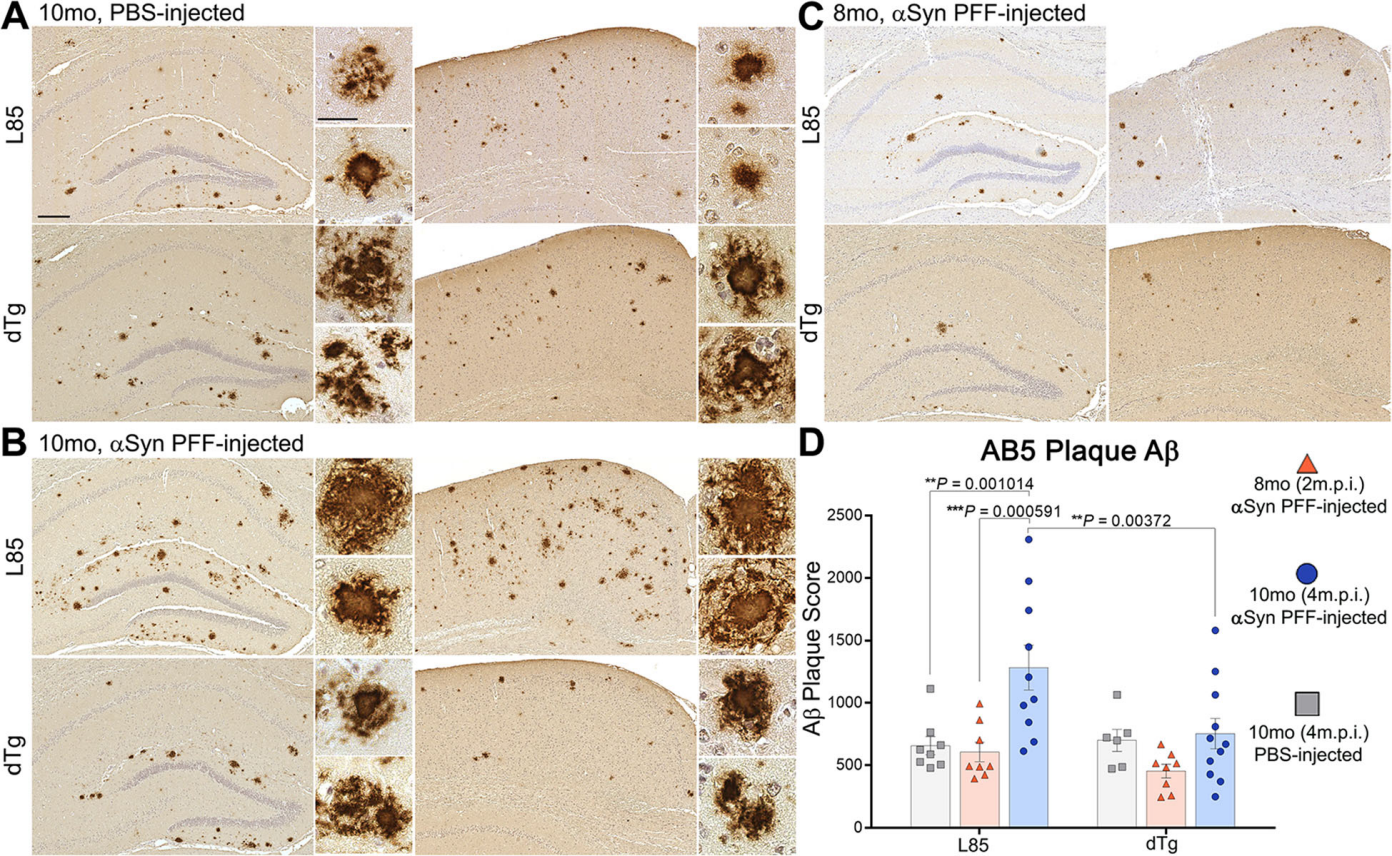
αSyn PFF-Injection Spurs Aβ Plaque Deposition in L85 but Not dTg Mice. **A-C** Representative images showing IHC using antibodies specific for Aβ (AB5), on sections from 10-month-old (4 m.p.i.) L85 and dTg mice injected with PBS (**A**), αSyn PFFs (**B**), and 8-month-old (2 m.p.i.) L85 and dTg mice injected with αSyn PFFs (**C**). **D** Grouped scatter plots depicting semi-quantitative analysis of AB5-positive plaques comparing L85 and dTg mice from 8-month and 10-month aged cohorts. Two-Way ANOVA followed by Holm-Sidak’s multiple comparisons (*n* = 8,8,10; 6,8,11). Data are presented as mean +/− SEM. Scale bars: high magnification = 200 μm; low magnification = 25 μm

In order to test whether αSyn PFF-injection and pathology modulated the spread and severity of Aβ plaques, we conducted an analysis on all cohorts of mice using an antibody that detects Aβ deposits (AB5). nTg and M20 cohorts did not exhibit plaque formation (Supplemental Figure 2), while both αSyn-injected and PBS-injected mice in the dTg and L85 cohorts showed extensive AB5-positive staining (Fig. 4A-C). Surprisingly, semi-quantitative analysis revealed that αSyn PFF-injection potentiated the accumulation of Aβ deposition in L85 mice, but not in dTg mice, despite dTg mice having extensive αSyn pathology (Fig. 4D). As expected for L85 mice, female mice tended to have a higher number of Aβ deposits relative to male mice, although by 10 months of age the difference was not statistically significant (Supplemental Figure 3). αSyn PFF injection appeared to increase Aβ deposition in both sexes of L85 mice (Supplemental Figure 3A). In the dTg mice, there was also a tendency for female mice to have higher numbers of deposits (Supplemental Figure 3B), but again, the difference was not statistically significant. The tendency for female dTg mice to have higher numbers of Aβ deposits was associated with a tendency for higher numbers of αSyn inclusions after PFF injection (Supplemental Figure 3C). Thus, although female L85 and dTg mice tended to have a greater burden of both Aβ and αSyn pathology, both sexes showed the same general response to injected αSyn PFFs.

### Neuroinflammatory changes associated with induced αSyn inclusion pathology

**Fig. 5:**
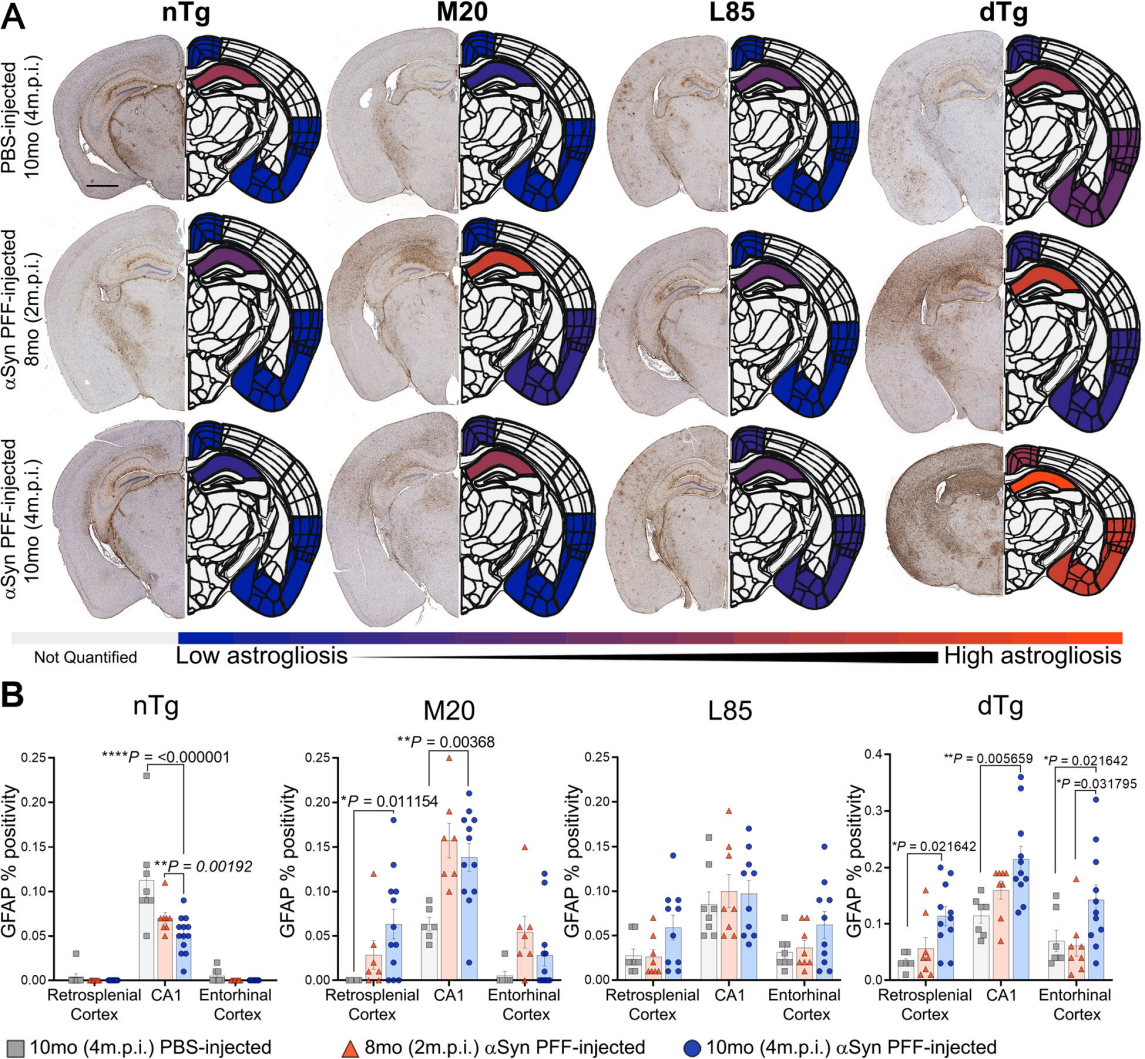
The Presence of Aβ and αSyn Pathology Induces More Severe Astrocytic Activation. **A** Representative images showing IHC using antibodies specific for GFAP to compare nTg, M20, L85, and dTg mice injected with PBS or αSyn PFFs at 8 (2 m.p.i.) and 10 months (4 m.p.i.) of age as indicated, and the corresponding heat map depicting regional GFAP percent positivity. The level of astrocytic activation is illustrated by the color change from blue (minimum of GFAP measured percent positivity) to orange (maximum measured GFAP percent positivity). Gray indicates regions were not quantified during this study. **B** Quantitation of GFAP percent positivity compares the intensity of activated astrocytes between the retrosplenial cortex, CA1 of the hippocampus, and the entorhinal cortex within each cohort. Two-Way ANOVA followed by Holm-Sidak’s multiple comparisons test was used for statistical analysis (*n* = 8,8,13; 6,8,12; 8,8,10; 6,8,11). Data are presented as mean +/− SEM. Scale bar: 1000 μm

**Fig. 6:**
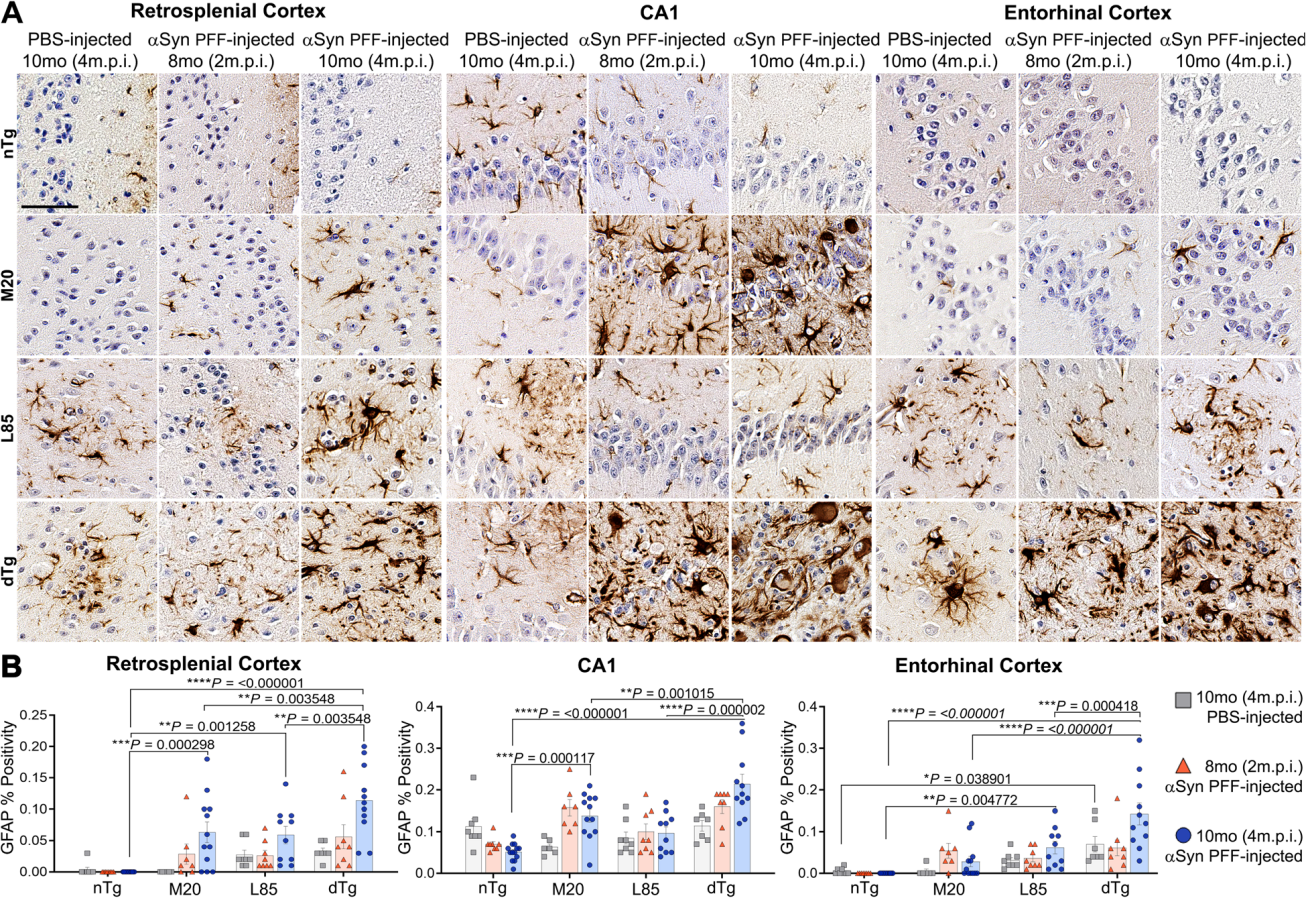
Extent of Induced Astrogliosis is Regionally Distinct. **A** Representative high magnification images showing IHC using antibodies specific for GFAP to compare regional astrogliosis in nTg, M20, L85, and dTg mice injected with PBS or αSyn PFFs at 8 (2 m.p.i.) and 10 months (4 m.p.i.) of age as indicated. **B** Quantitation of GFAP percent positivity compares the intensity of activated astrocytes in the retrosplenial cortex, CA1 of the hippocampus, and the entorhinal cortex between each cohort. Two-Way ANOVA followed by Holm-Sidak’s multiple comparisons test (*n* = 8,8,13; 6,8,12; 8,8,10; 6,8,11). Data are presented as mean +/− SEM. Scale bar: 50 μm

**Fig. 7:**
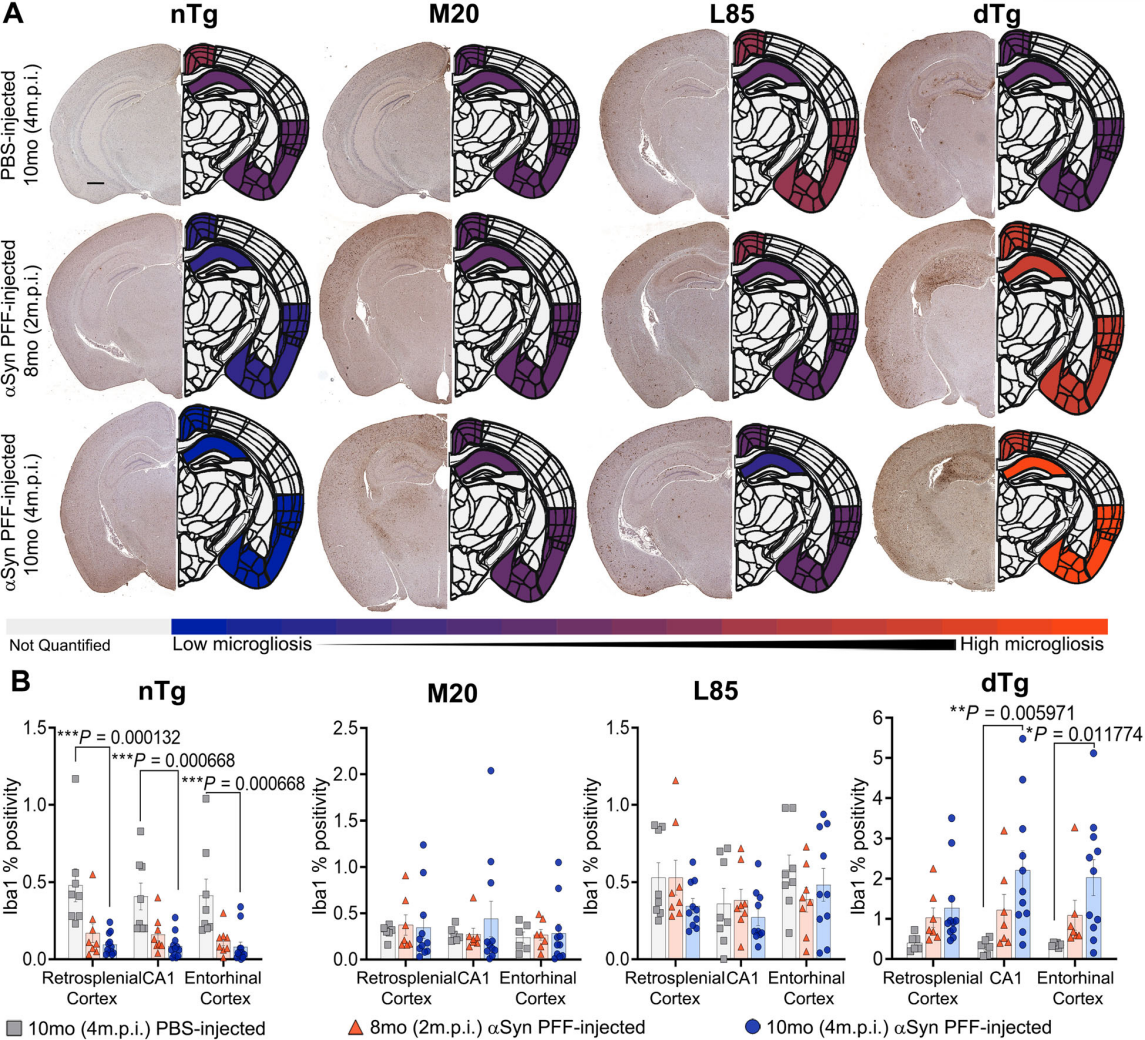
Exacerbation of Microgliosis in αSyn PFF-seeded dTg mice. **A** Representative images showing IHC using antibodies specific for Iba1 to compare nTg, M20, L85, and dTg mice injected with PBS or αSyn PFFs at 8 (2 m.p.i.) and 10 months (4 m.p.i.) of age as indicated, and corresponding heatmap depicting regional Iba1 percent positivity. The increase in microglial proliferation is illustrated by the color change from blue (minimum of Iba1 percent positivity) to orange (maximum of Iba1 percent positivity). Gray indicates regions were not quantified during this study. **B** Quantitation of Iba1 percent positivity comparing the retrosplenial cortex, CA1 of the hippocampus, and the entorhinal cortex within each cohort. Two-Way ANOVA followed by Holm-Sidak’s multiple comparisons test was used for statistical analysis (*n* = 8,8,13; 6,8,12; 8,8,10; 6,8,11). Data are presented as mean +/− SEM. Scale bar: 500 μm

**Fig. 8:**
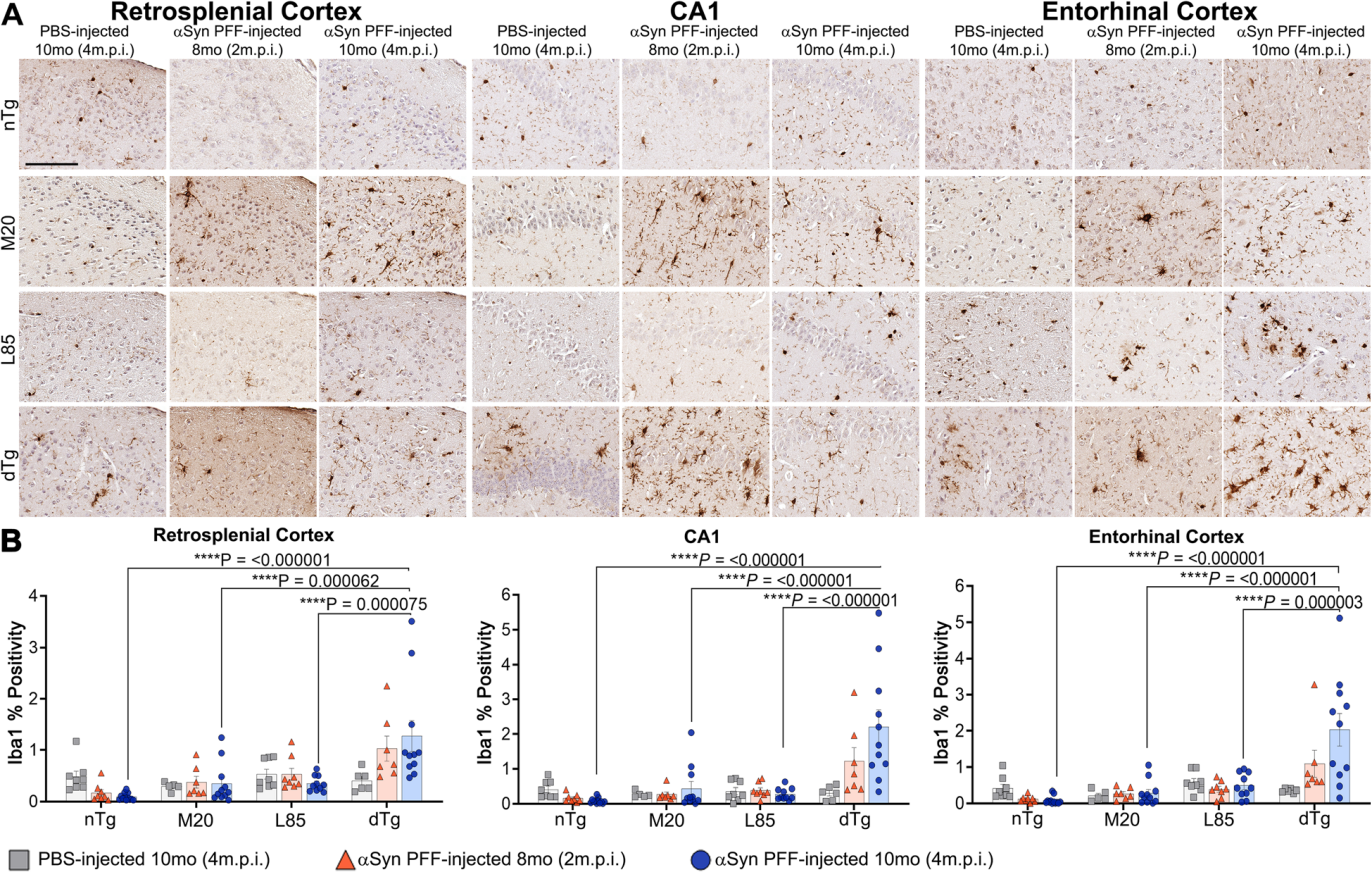
Microglial Activation Increases in a Parallel Pattern Across Regions and Follows Escalation of αSyn and Aβ Pathology. **A** Representative high magnification images showing IHC using antibodies specific for Iba1, to compare regional microglial activation in nTg, M20, L85, and dTg mice injected with PBS or αSyn PFFs at 8 (2 m.p.i.) and 10 months (4 m.p.i.) of age as indicated. **B** Quantitation of Iba1 percent positivity comparing the retrosplenial cortex, CA1 of the hippocampus, and the entorhinal cortex between each cohort. Two-Way ANOVA followed by Holm-Sidak’s multiple comparisons test (*n* = 8,8,13; 6,8,12; 8,8,10; 6,8,11). Data are presented as mean +/− SEM. Scale bar: 50 μm

To provide further insights into the pathological changes associated with prion-like induced αSyn inclusion pathology and Aβ deposition, changes in astrogliosis (GFAP) (Figs. 5 and 6) and microgliosis (Iba1) (Figs. 7 and 8) were investigated. Dramatic differences in glial activation responses were observed between different regions. To accommodate this regional variability, we segmented quantification into separate analyses of the retrosplenial cortex, CA1 region of the hippocampus and the combined entorhinal/piriform cortex with the amygdalar region (Figs. 6 and 8).

In the nTg mice, GFAP reactivity was very low in the retrosplenial or entorhinal cortex (Fig. 5B). Surprisingly, nTg mice injected with PFFs displayed lower GFAP percent positivity in the CA1 region than age-matched, PBS-injected controls (Fig. 5B). M20 mice injected with αSyn PFF displayed increased astrogliosis in all brain regions examined (Figs. 5B and 6B). In L85 mice, regardless of whether injected with PFFs or PBS, activated astrocytes were primarily located adjacent to Aβ deposits. By contrast, in the dTg mice, PFF injection induced astrocytic activation that was significantly more severe than age-matched mice in L85, M20 and nTg cohorts; in all regions that were measured (Figs. 5B and 6B). Because the level of Aβ pathology in the dTg mice injected with PFFs was lower than that of the L85 mice (Fig. 4C), we conclude that the more severe astrocytic response in the dTg mice was primarily driven by the induced αSyn pathology.

To examine microgliosis reactions, we stained sections with Iba1 antibodies. Overall, Iba1 immunoreactivity patterns paralleled that of GFAP (Figs. 7 and 8). In both the CA1 and entorhinal regions, microgliosis was significantly increased in 10-month dTg injected with αSyn PFFs compared to the PBS injected cohort. Similarly, as compared to nTg, L85, and M20 mice, Iba1 reactivity was more widespread in the dTg mice injected with PFFs (Figs. 7 and 8). These increases were observed in all three regions examined (retrosplenial cortex, CA1 hippocampal, and entorhinal cortex). Neither M20 nor L85 mice had a significant change in microglial response with injection type or aging despite exhibiting an increased protein aggregate burden (see Figs. 2C and 4D, respectively). Some of the Iba1 immunoreactivity may be marking infiltrating peripheral monocytes. Attempts to differentiate such cells with immune markers, such as TMEM119 and CD68, were unsuccessful due to weak immunoreactivity for these proteins in our samples. The poor performance of the antibodies may be related to the ethanol fixation methods we use here to preserve certain αSyn epitopes.

## Discussion

Our study has shown that the deposition of Aβ in the cortex and hippocampus creates an environment in which human αSyn pathology spreads more quickly and distributes across a greater area following prion-like seeding. The model created by our approach exhibits inclusions composed of WT human αSyn and human Aβ plaque pathology, allowing us to investigate the interactive sequelae associated with the progression of both types of protein aggregations. We observe an exacerbated inflammatory response in mice exhibiting both Aβ and αSyn pathology as compared to brains depositing these proteins individually. An important aspect of our model is that M20 mice do not develop αSyn pathology sans seeding, allowing us to delay the induction of synucleinopathy until after Aβ pathology had developed. At the ages that we examined, no αSyn pathology was observed in dTg mice injected with PBS, indicating that the induced αSyn pathology was highly dependent upon seeding. Furthermore, human αSyn PFF injection did not induce pathology in nTg or L85 mice. Collectively, our findings demonstrate that prion-like propagation of human αSyn pathology spreads more quickly when induced in the presence of pre-existing Aβ pathology to produce a model that resembles human LBD.

Our findings are consistent with a recent study where mouse αSyn PFFs were injected in the 5xFAD model of Aβ deposition, finding a dramatic induction of mouse αSyn pathology when seeds were injected at ages in which pre-existing amyloid pathology was present [60]. The location of αSyn deposition in both models is remarkably similar, possibly related to the similarity in the distribution of Aβ pathology in the L85 and 5xFAD mice. Notably, human αSyn PFFs did not induce mouse αSyn pathology in our L85 mice; a finding that is consistent with the premise of a ‘species barrier’ for prion-like proteins; a concept that hinges on the idea that human PFFs serve as a “seed” able to induce monomers in a solution to assume a β-sheet conformation, and eventual fibril elongation [61, 62], but require the appropriate secondary structural compatibilities for efficient conformational templating to occur [37, 63–65]. Human αSyn forms different quaternary structures than mouse αSyn, and this presumably inhibits the conformational templating of mouse αSyn [37, 66].

Previous studies in bigenic APP and αSyn mice had reported that Aβ deposition could exacerbate αSyn pathology without seeding [67]. In our dTg model, at the ages examined, we did not observe human αSyn inclusion pathology without seeding. The contrasting outcomes may be due to nature of the transgene expression or strains of mice. The effect that αSyn has on the deposition of Aβ has been examined in multiple studies with some studies demonstrating inhibitory activities of αSyn on Aβ plaque formation [68] and others the opposite [69, 70]. For example, Clinton et al. crossed 3xTg-AD mice, which develop Aβ and tau pathology [71], with the M83 model of A53T synucleinopathy [35], finding mutant αSyn promoted Aβ aggregation [69]. In 2018, Khan et al. [70] published data suggesting that the levels of αSyn overexpression are inversely correlated with the amount of Aβ plaque accumulation in J20 APP transgenic mice crossed with TgI2.2 mice, which overexpress WT human αSyn. These studies reflect the initial condition dependent nature of Aβ and αSyn interactions. Clinton et al. [69] used hemizygous M83 mice, which develop αSyn pathology at 22–28 months [35], but reported seeing an increase in thioflavin S positive Aβ plaques at 12 months, before αSyn pathology would be present. In the second study mentioned, Khan et al. specifically measured a difference in amyloid burden during the preliminary stages of plaque deposition (aged animals to 6 month), comparing early amyloid deposition in the hippocampus [70]. Therefore, both of these studies reported potential effects of non-aggregated αSyn on initial Aβ plaque deposition.

In our cohorts of dTg (L85/M20) mice we found no obvious difference in Aβ burden between dTg and L85 mice injected with PBS. The L85 model primarily presents with cored-neuritic deposits of Aβ and thus it would appear that this type of Aβ pathology is not greatly influenced by the presence of elevated levels of WT human αSyn. Similar to what was reported in 5xFAD mice injected with mouse αSyn PFFs [60], αSyn PFF-injection in L85 mice appeared to increase plaque burden. By contrast, in the dTg mice, the injection of PFFs did not produce the same augmentation in Aβ pathology; however, we observed increased levels of both microglial and astrocytic activation after PFF injection within this cohort when compared to PBS-injected dTg mice. Many studies have emphasized the importance of gliosis in the attenuation of Aβ plaque deposition. Previous work by Chakrabarty et al. has shown that decreased neuroinflammation, brought on by anti-inflammatory cytokines, such as Interleukin-10 or Interleukin-4, suppresses microglial phagocytosis of Aβ plaques and worsens cognitive deficits in APP mice [72, 73], whereas upregulation of the proinflammatory cytokines, Interleukin-6, Interferon γ or Tumor Necrosis Factor α, results in a reduction of Aβ plaque deposition [52, 74, 75]. Shaftel et al. demonstrated that hippocampal overexpression of the proinflammatory cytokine, IL-1β, results in a reduction of amyloid pathology in APP mice [76]. Taken together, these data demonstrate a complicated interplay between different pathologies that appear to influence the overall evolution of pathology.

As αSyn was initially described as the non-amyloid component of amyloid plaques (NACP) [77], the direct interactions and copolymerization of αSyn and Aβ have been reported in many in vitro studies [67, 78–83]. In fact, PFFs have been shown to be able to nucleate Aβ aggregation [84]; however the literature on this topic is complicated. In 2020, Candreva et al., demonstrated that αSyn monomers and oligomers co-assemble with Aβ, stabilizing Aβ oligomers and thus preventing Aβ fibrillization, whereas αSyn fibrils did not change fibrillization [83]. Furthermore, the effect of αSyn on Aβ fibrilization was lost when aggregation studies were seeded with preformed Aβ fibrils [83]. Taken together, these results hint towards a possible sequence-dependent phenomenon, where progression of pathology depends on which protein first began forming pathological inclusions. The process is further complicated by inflammatory changes that each type of pathology may also induce.

The ability of αSyn and Aβ to copolymerize suggests a potential mechanism in which pre-existing Aβ pathology could augment αSyn seeding. It is possible that αSyn PFFs are able to directly interact with Aβ deposits at the time of injection to stabilize the αSyn seeds in a manner that potentiates seeding. While it is conceivable that these exogenous αSyn PFFs were able to seed additional Aβ plaques in L85 mice, our measurements, recorded at the end stage of the disease, did not detect an obvious concentration of αSyn inclusions near Aβ deposits. It is also possible that neuronal hyperactivation resulting from, or even preceding, Aβ plaque formation in APP mouse models [85–87], promoted PFF neuronal uptake. Elevated neuronal activity can significantly influence neuronal αSyn cellular uptake and release [88, 89]. Consistent with this notion, Wu et al. [90] recently demonstrated that increasing neuronal activity in hippocampal and midbrain slice cultures from 5xFAD mice treated with αSyn PFFs enhanced seeding of αSyn inclusions.

## Conclusions

Clinical evidence points to a preponderance of co-pathologies between prionogenic proteins that are correlated to neurodegenerative diseases [10, 11, 13–16, 18, 28], however the cause-effect relationships are difficult to ascertain in human post-mortem studies, and is better determined using animal experimental models. Nevertheless, the human genetic and pathological findings that patients with genetic alterations in the *APP*, *PSEN1,* and *PSEN2* genes that drive Aβ deposition, also predispose patients to develop αSyn pathology, provide strong evidence of collusion between aberrant Aβ accumulation and αSyn, in the pathobiology of neurodegenerative diseases [12, 24–27]. This clinical revelation is rapidly being translated into animal models for further exploration, with many research teams developing multi-malprotein overexpression models to analyze how different combinations of pathological proteins can affect the progression of disease [60, 67–69, 91, 92]. Therefore, our intention in this study was primarily to create a novel and accurate mouse model that closely capitulates authentic conditions in neurodegenerative diseases. In summary, we present a humanized model of AD/LBD, in which pre-existing Aβ deposition augments the seeding activity of human αSyn PFFs to produce pathology resembling AD/LBD. Our novel model provides a new platform to examine pathogenic protein interactions between human αSyn and Aβ, and the in vivo assessment of therapeutic interventions.

### Supplementary Information

**Additional file 1: Supplemental Figure 1.** L85 and nTg Mice do not Exhibit αSyn Pathology Despite αSyn PFF-injection. Representative images showing immunohistochemistry of tissue sections stained using antibodies specific for phosphorylated αSyn (81A), αSyn (2H6) and p62/sequestrasome-1 from αSyn PFF-injected nTg and αSyn PFF-injected L85 mice in the 10-month-old (4 m.p.i.) cohort. No inclusions of endogenous αSyn were observed in these cohorts. Scale bar: 100 μm. The images shown are representative of independent IHC stains from all animals. **Supplemental Figure 2.** M20 and nTg Mice do not Exhibit Aβ Pathology. Representative images showing IHC using antibodies specific for Aβ (AB5), on sections from 10-month-old (4 m.p.i.) αSyn PFF-injected nTg and M20 mice. No Aβ plaque pathology was observed in these cohorts. Scale bar: 200 μm. The images shown are representative of independent IHC stains from all animals. **Supplemental Figure 3.** Analysis of Aβ and αSyn pathology by sex. Semi-quantitative data for Aβ deposits and αSyn pathology levels in 10-month-old L85 and dTg mice are graphed according to sex. Female, PBS-injected L85 and dTg mice tended to have higher numbers of Aβ deposits **(A-B).** There was no statistically significant difference between males and females with either Aβ plaque deposition or αSyn inclusion pathology **(A-C)**; therefore, all quantitative analyses combined data from both sexes.

## Data Availability

The datasets used and analyzed during the current study are available from the corresponding author on reasonable request.

## Abbreviations

AD: Alzheimer’s Disease
APP: Amyloid precursor protein
Aβ: Amyloid beta
αSyn: Alpha-synuclein
CNS: Central nervous system
CSF: Cerebrospinal fluid
dTg: Double transgenic
GFAP: Glial fibrillary acidic protein
HIER: Heat-induced epitope retrieval
IRA: Immunoreactive Area
Iba1: Ionized calcium binding adaptor molecule 1
IFA: Immunofluorescence analysis
IFN-γ: Interferon-γ
IHC: Immunohistochemistry
IL-10: Interleukin-10
IL-6: Interleukin-6
LB: Lewy bodies
LBD: Lewy Body Dementia
LN: Lewy neurites
m.p.i.: Months post-injection
MSA: Multiple Systems Atrophy
NDDs: Neurodegenerative disorders
NFL: Neurofilament light chain
nTg: Non-transgenic
OD: Optical Density
PD: Parkinson’s disease
PDD: Parkinson’s Disease with Dementia
PFF: Preformed fibrils
PS1: Presenilin 1
SNpc: Substantia Nigra pars compacta
SQSTM1: Sequestrasome1
TNF α: Tumor Necrosis Factor α
