# Correction: Collusion of α-Synuclein and Aβ aggravating co-morbidities in a novel prion-type mouse model

**DOI:** 10.1186/s13024-024-00710-2

**Published:** 2024-02-16

**Authors:** Grace M. Lloyd, Jess-Karan S. Dhillon, Kimberly-Marie M. Gorion, Cara Riffe, Susan E. Fromholt, Yuxing Xia, Benoit I. Giasson, David R. Borchelt

**Affiliations:** 1 Department of Neuroscience, College of Medicine, University of Florida, Gainesville, FL 32610 USA; 2 Center for Translational Research in Neurodegenerative Disease, College of Medicine, University of Florida, Gainesville, FL 32610 USA; 3 McKnight Brain Institute, College of Medicine, University of Florida, Gainesville, FL 32610 USA

**Correction: Molecular Neurodegeneration 16, 63 (2021)**

**https://doi.org/10.1186/s13024-021-00486-9**

The authors wish to correct an error in Fig. 7A of the original article [1]. We have determined that the representative image of a hemibrain shown for 10 month-old, PBS-injected, nontransgenic (nTg) mice in panel A is incorrect. Due to a labeling error of the digital file name, the brain image shown was actually from a 10 month-old, PBS-injected, wild-type αSyn (M20) animal. The corrected panel for Fig. 7A is provided in the figure shown below. It is imperative to emphasize that this correction does not alter the conclusions presented in the paper. None of the 10 month-old nTg or M20 mice injected with PBS exhibited appreciable pathology.

**Fig. 7:**
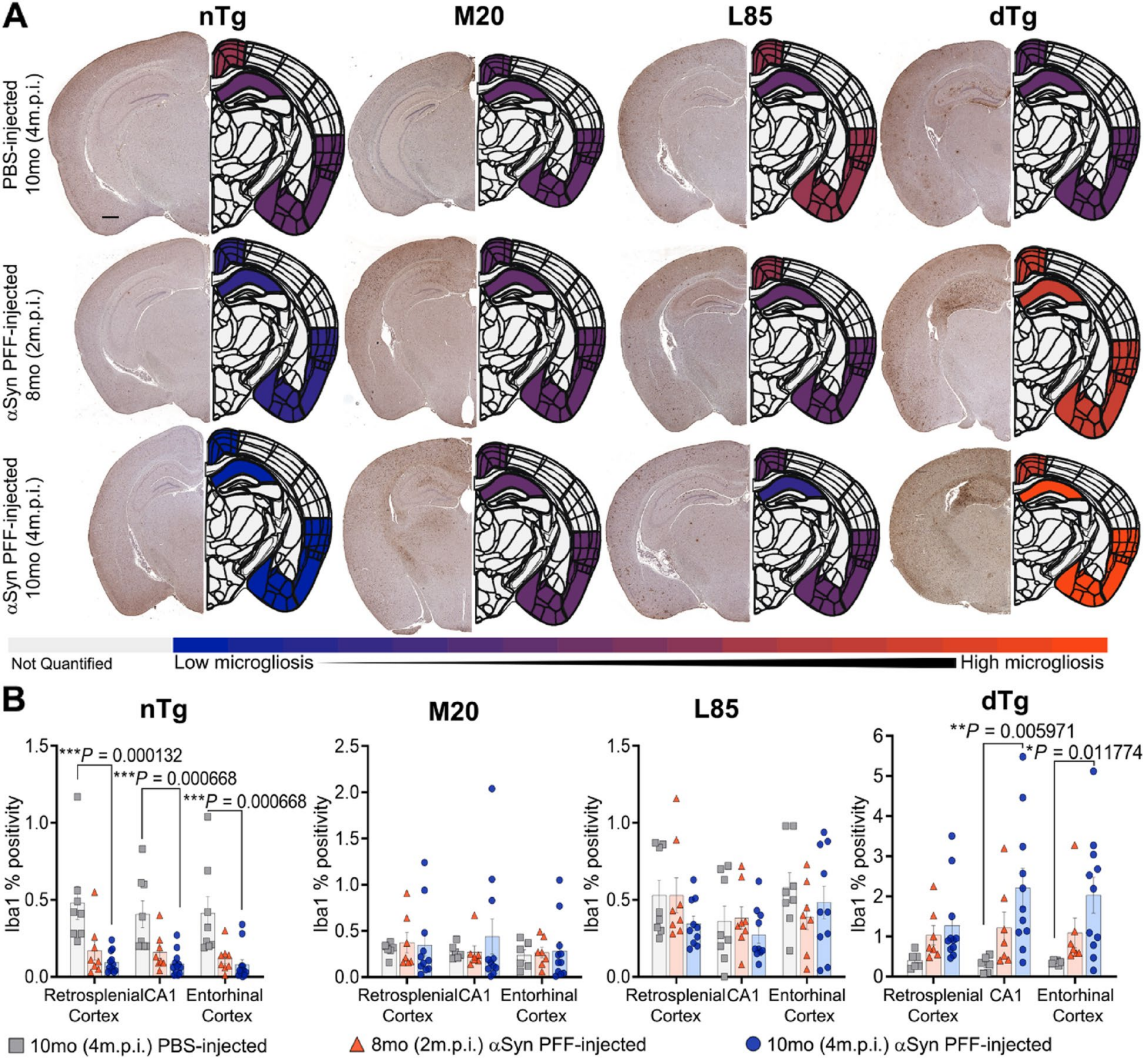
Exacerbation of Microgliosis in αSyn PFF-seeded dTg mice. **A** Representative images showing IHC using antibodies specific for Iba1 to compare nTg, M20, L85, and dTg mice injected with PBS or αSyn PFFs at 8 (2 m.p.i.) and 10 months (4 m.p.i.) of age as indicated, and corresponding heatmap depicting regional Iba1 percent positivity. The increase in microglial proliferation is illustrated by the color change from blue (minimum of Iba1 percent positivity) to orange (maximum of Iba1 percent positivity). Gray indicates regions were not quantified during this study. **B** Quantitation of Iba1 percent positivity comparing the retrosplenial cortex, CA1 of the hippocampus, and the entorhinal cortex within each cohort. Two-Way ANOVA followed by Holm-Sidak’s multiple comparisons test was used for statistical analysis (*n* = 8,8,13; 6,8,12; 8,8,10; 6,8,11). Data are presented as mean + / − SEM. Scale bar: 500 μm

The authors sincerely regret any confusion this oversight may have caused and appreciate the understanding of the readership.
