# Supplementary material for: Collusion of α-Synuclein and Aβ aggravating co-morbidities in a novel prion-type mouse model
Source: Mol Neurodegener. 2021 Sep 9;16:63. doi: 10.1186/s13024-021-00486-9 (PMC8427941; doi:10.1186/s13024-021-00486-9)
Supplement: Supplementary file 1 — Additional file 1: Supplemental Figure 1. L85 and nTg Mice do not Exhibit αSyn Pathology Despite αSyn PFF-injection. Representative images showing immunohistochemistry of tissue sections stained using antibodies specific for phosphorylated αSyn (81A), αSyn (2H6) and p62/sequestrasome-1 from αSyn PFF-injected nTg and αSyn PFF-injected L85 mice in the 10-month-old (4 m.p.i.) cohort. No inclusions of endogenous αSyn were observed in these cohorts. Scale bar: 100 μm. The images shown are representative of independent IHC stains from all animals. Supplemental Figure 2. M20 and nTg Mice do not Exhibit Aβ Pathology. Representative images showing IHC using antibodies specific for Aβ (AB5), on sections from 10-month-old (4 m.p.i.) αSyn PFF-injected nTg and M20 mice. No Aβ plaque pathology was observed in these cohorts. Scale bar: 200 μm. The images shown are representative of independent IHC stains from all animals. Supplemental Figure 3. Analysis of Aβ and αSyn pathology by sex. Semi-quantitative data for Aβ deposits and αSyn pathology levels in 10-month-old L85 and dTg mice are graphed according to sex. Female, PBS-injected L85 and dTg mice tended to have higher numbers of Aβ deposits (A-B). There was no statistically significant difference between males and females with either Aβ plaque deposition or αSyn inclusion pathology (A-C); therefore, all quantitative analyses combined data from both sexes. [file 13024_2021_486_MOESM1_ESM.pdf]

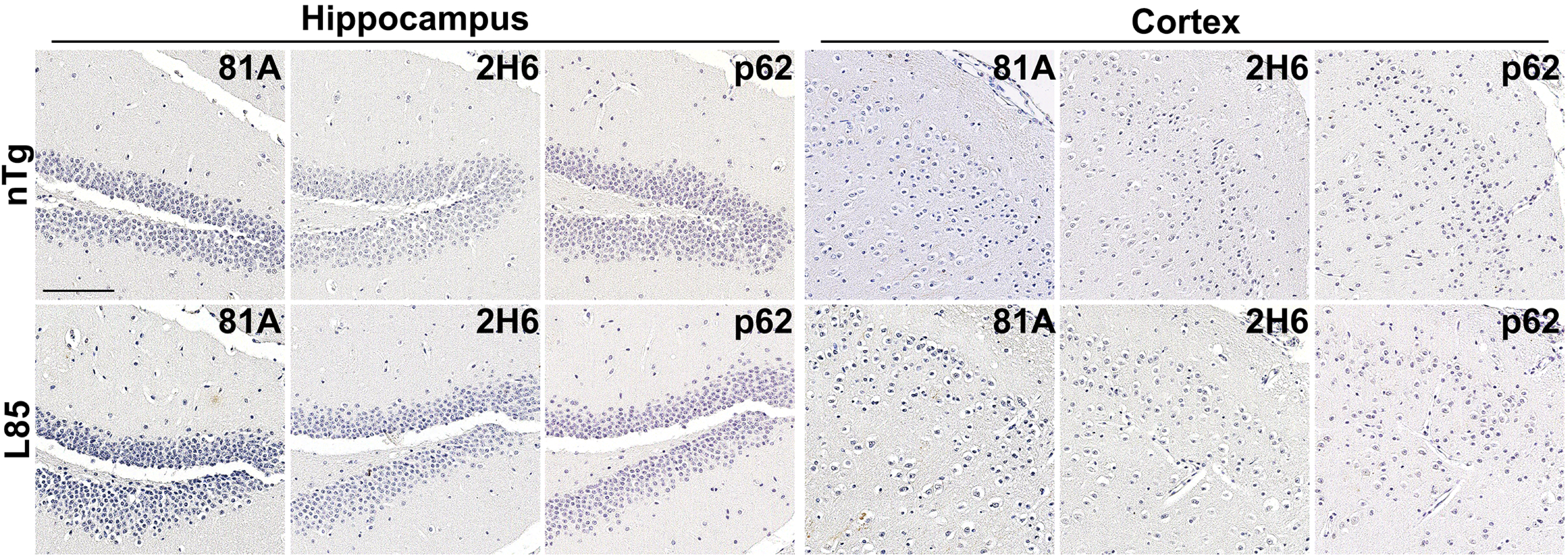

Supplemental Figure 1

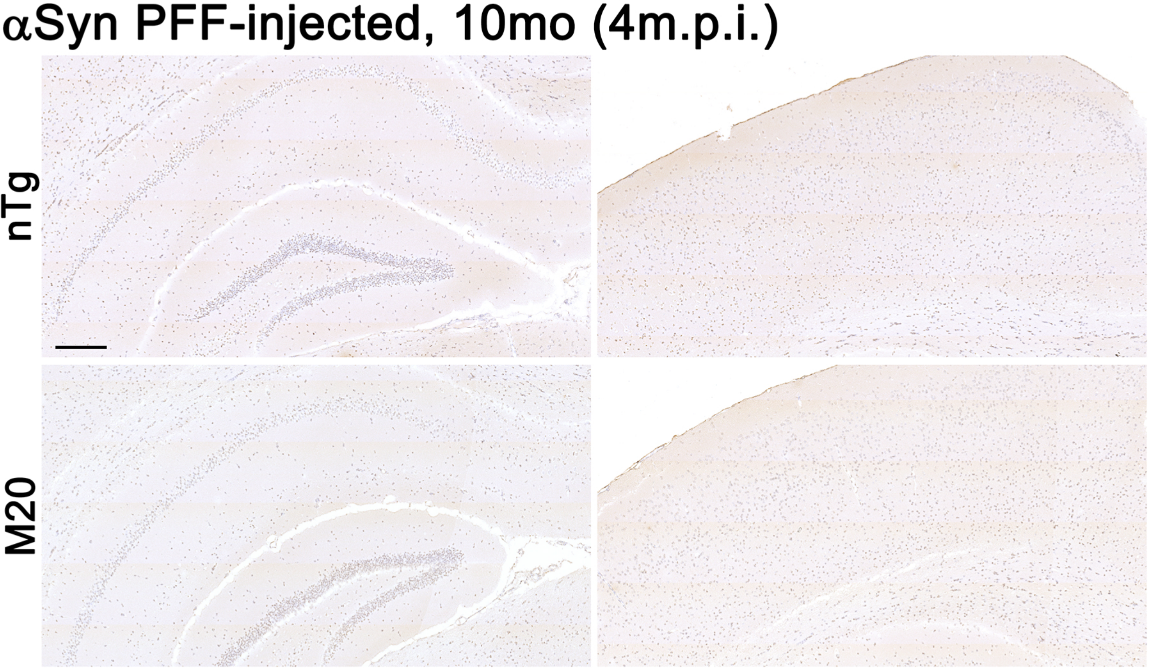

Supplemental Figure 2

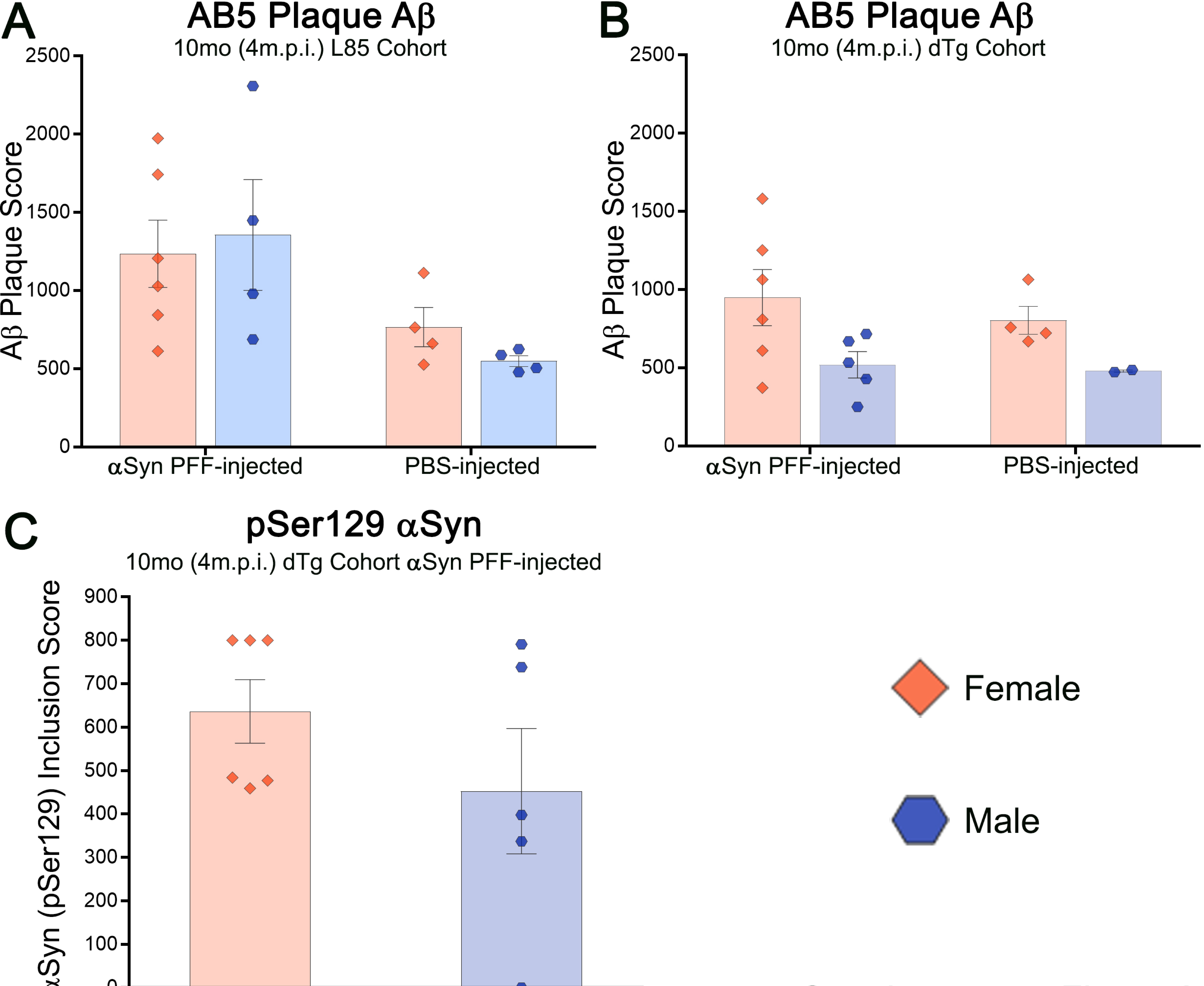

Supplementary Figure 3
